# Triple immunotherapy containing telitacicept for systemic lupus erythematosus complicated by multiple autoimmune diseases: a case report

**DOI:** 10.3389/fimmu.2026.1951267

**Published:** 2026-09-24

**Authors:** Yuqing Ge, Aifei Zhang, Longyan Qin, Didi Wang, Jiashun Zeng

**Affiliations:** Department of Rheumatology and Immunology, The Affiliated Hospital of Guizhou Medical University, Guiyang, China

**Keywords:** BLyS/APRIL, immune-mediated necrotizing myopathy, psoriasis, Sjögren’s syndrome, systemic lupus erythematosus, telitacicept

## Abstract

SLE may occur simultaneously with or subsequently to other autoimmune diseases; such cases are called overlap syndromes. Cases of SLE overlapping with three or more autoimmune diseases are extremely rare, and there is no unified standard or consensus for treatment. We report a clinical case of a patient with SLE, immune-mediated necrotizing myopathy, Sjögren’s syndrome, and psoriasis. After treatment with a triple immunosuppressive regimen containing telitacicept, the patient achieved a satisfactory therapeutic response. As the first dual-pathway inhibitor targeting BLyS/APRIL, telitacicept demonstrated potential in the treatment of this complex overlap syndrome, suggesting a promising application prospect in patients with multiple coexisting autoimmune diseases.

## Introduction

Systemic lupus erythematosus (systemic lupus erythematosus, SLE) is a typical autoimmune connective tissue disease, characterized by inflammatory responses and immune-mediated damage involving multiple organ systems (1). It often presents clinically with systemic symptoms such as fever, skin erythema, photosensitivity, and arthralgia, and may be accompanied by involvement of the kidneys, blood, nervous system, serosa, and other organs or systems. Treatment of SLE is stratified according to disease severity. Mild SLE is mainly treated with low-dose glucocorticoids and hydroxychloroquine. Severe SLE (such as lupus nephritis and neuropsychiatric lupus) is treated in two phases: induction of remission and maintenance therapy. The induction phase aims to rapidly control active lesions, with the core regimen being glucocorticoids combined with cyclophosphamide or mycophenolate mofetil. The maintenance phase uses mycophenolate mofetil or azathioprine for long-term treatment while gradually tapering glucocorticoids. At the same time, multiple guidelines have incorporated biologics, such as belimumab and CD20 monoclonal antibodies, into treatment recommendations for severe SLE (2–4).

SLE may occur simultaneously with or after other autoimmune diseases; such cases are known as overlap syndromes. SLE complicated by rheumatoid arthritis, Sjögren’s syndrome, systemic sclerosis, and inflammatory myopathy is relatively common (5). However, cases overlapping with three or more autoimmune diseases are extremely rare, and treatment lacks unified standards and consensus. This article reports a clinical case of a patient with SLE, immune-mediated necrotizing myopathy, Sjögren’s syndrome, and psoriasis who was treated with a triple immunotherapy regimen containing telitacicept, with the aim of summarizing the diagnostic and therapeutic experience and providing a reference for the clinical management of similar complex autoimmune overlap cases.

## Case presentation

A 46-year-old man presented to the dermatology department of a hospital in October 2021 because of “facial and limb rash with itching for several months.” The rash consisted of several 2–5 mm × 2–5 mm erythematous maculopapules on the face, limbs, back, and both ears, some of which were confluent and covered with silvery-white scales. Pathological biopsy confirmed the diagnoses of vulgar psoriasis and erythematosus lupus. He was treated with hydroxychloroquine 0.2 g bid (later discontinued due to drug-induced arrhythmia), compound glycyrrhizin tablets 50 mg tid, and triamcinolone acetonide econazole cream, after which the desquamation improved.

In January 2022, the patient was hospitalized in the neurology department of a hospital for “right-sided limb weakness with loss of consciousness and generalized convulsions for more than 1 week.” Laboratory tests showed ANA 1:1000, strongly positive anti-nRNP/Sm antibodies, positive anti-SSA antibodies, strongly positive anti-SSA 52, and positive anti-histone antibodies. Cerebrospinal fluid showed protein qualitative 1+, total protein 582 mg/L, and no evidence of infection. Autoimmune encephalitis antibodies, antineutrophil cytoplasmic antibodies, anti-double-stranded DNA antibodies, and anti-Sm antibodies were all negative. Contrast-enhanced cranial MRI showed swelling and abnormal signal in the left superior frontal gyrus and part of the precentral gyrus cortex, abnormal enhancement of the left parietal dura mater, suggesting possible cortical venous thrombosis, with infectious lesions to be excluded. EEG reported an abnormal electroencephalogram with sharp slow waves in the right frontal and anterior temporal regions. Electromyography suggested sensory peripheral nerve damage. Lip gland biopsy was consistent with Sjögren’s syndrome (grade IV). Considering Sjögren’s syndrome with nervous system involvement and symptomatic epilepsy, the patient was given methylprednisolone 1 g/day for 3 consecutive days as pulse therapy, followed by 60 mg qd, combined with mycophenolate mofetil 0.25 g bid for immunomodulation. After discharge, glucocorticoids were gradually tapered, and the final stable regimen was prednisone 2.5 mg qd combined with mycophenolate mofetil 0.25 g qd.

In April 2025, the patient was treated at our center because of “limb weakness and pain in both hands’ joints for more than 2 weeks.” On admission, vital signs were stable. Several brown to light-red papules measuring 5 mm–1 cm × 5 mm–1.5 cm with relatively clear borders were seen on the limbs and back, some surfaces covered with silvery-white scales. Both hands were swollen, with tenderness and swelling of the metacarpophalangeal joints. No special findings were noted on cardiopulmonary or abdominal examination. Limb muscle strength was grade 3, and pathological signs were negative. Laboratory findings were as follows: white blood cell count 2.10 × 10^9^/L, absolute neutrophil count 1.26 × 10^9^/L, hemoglobin 88.00 g/L; erythrocyte sedimentation rate 29.88 mm/h, C-reactive protein 10.92 mg/L; alanine aminotransferase 103.80 U/L, aspartate aminotransferase 397.10 U/L; antinuclear antibody titer 1:1000, weakly positive anti-nRNP/Sm antibody, strongly positive anti-SS-A52 antibody, positive anti-Ro-52 antibody, weakly positive anti-mitochondrial M2 antibody, anti-cyclic citrullinated peptide antibody 54.00 AU/mL, negative anti-double-stranded DNA antibody, anti-Sm antibody, antineutrophil cytoplasmic antibody, and anticardiolipin antibody, complement C3 0.296 g/L, complement C4 0.073 g/L, immunoglobulin G 27.80 g/L, direct antiglobulin test positive (C3d subtype); creatine kinase 5439.00 U/L, myoglobin 1870.00 ng/mL, troponin I 0.041 ng/mL; myositis antibody profile: anti-SRP antibody positive; urinalysis showed urine occult blood 1+, urine protein 1+, and 24-hour urine protein quantification of 722.80 mg; echocardiography showed a small amount of pericardial effusion; chest CT showed interstitial changes in both lungs, enlarged mediastinal and axillary lymph nodes, and splenomegaly; MRI of both hands showed multiple bone marrow edema in the carpal bones and soft tissue swelling; electromyography suggested peripheral nerve damage. Renal biopsy: focal proliferative and sclerosing lupus nephritis class III (A/C); according to the 2019 EULAR/ACR SLE classification criteria, the score was 23 points, meeting the diagnostic criteria for SLE; for markedly elevated muscle enzymes with anti-SRP antibody positivity (the patient refused muscle biopsy), according to the 2017 EULAR/ACR classification criteria for idiopathic inflammatory myopathy, immune-mediated necrotizing myopathy was considered. Based on the previous medical history and the current clinical presentation and auxiliary examinations, the patient was diagnosed with overlap syndrome of systemic lupus erythematosus, lupus nephritis, hematologic involvement, lupus encephalopathy, Sjögren’s syndrome, immune-mediated necrotizing myopathy, and psoriasis.

Treatment consisted of methylprednisolone 250 mg qd for 3 days of pulse therapy; after the pulse, the regimen was changed to methylprednisolone 40 mg once daily, combined with cyclophosphamide 0.5 g every 2 weeks × 6 and telitacicept 160 mg once weekly for intensified immunosuppression. After discharge, the patient received regular treatment. Four weeks later, the rash had subsided, creatine kinase decreased to 3577 U/L, and C3, C4, CRP, and 24-hour urine protein all returned to the normal range. At 8 weeks of treatment, the rash had almost disappeared, and creatine kinase further decreased to 798 U/L. At 12 weeks of treatment, creatine kinase decreased to 120 U/L, muscle strength recovered to grade 5, the cumulative dose of cyclophosphamide reached 3 g, and maintenance therapy was sequentially changed to mycophenolate mofetil 0.5 g orally twice daily, while telitacicept 160 mg once weekly was continued. At the 24-week follow-up, methylprednisolone had been tapered to 4 mg, all indicators remained normal continuously (urine protein negative, no hypocomplementemia, creatine kinase, CRP, and ESR normal), and no further symptoms such as rash, arthralgia, or limb weakness occurred; the disease was well controlled.

## Discussion

Systemic lupus erythematosus is a chronic systemic autoimmune disease characterized by abnormal activation of the immune system, production of large amounts of autoantibodies, formation of immune complexes, and subsequent inflammatory damage to multiple organs and tissues throughout the body (6). When SLE is complicated by multiple autoimmune diseases, diagnosis and treatment become extremely complex. This article reports a case of SLE complicated by lupus nephritis, hematologic involvement, and lupus encephalopathy, concurrently accompanied by immune-mediated necrotizing myopathy, Sjögren’s syndrome, and psoriasis. Satisfactory therapeutic results were achieved after regular treatment with a triple regimen containing telitacicept.

The pathogenesis of overlap syndrome has not yet been fully elucidated. It is currently believed to result from the combined effects of multiple factors such as genetic susceptibility, environmental triggers, and immune dysregulation. In terms of genetic susceptibility, the HLA-DR4 allele is associated with multiple diseases including rheumatoid arthritis and systemic lupus erythematosus (7), and variants in some genes involved in immune regulation, such as IRF5 and STAT4, have also been found to increase the risk of multiple autoimmune diseases simultaneously (8, 9). On this basis, certain environmental factors (such as EBV/bacterial infection) may act as “triggers” and induce cross-reactive immune responses against self-tissues through a “molecular mimicry” mechanism (10, 11). Immune dysregulation is the central link in the development of overlap syndrome, with overactivation of B cells and excessive autoantibody production being the most prominent features. On the one hand, B-cell tolerance is breached at two levels: centrally, defects in B-cell receptor editing in the bone marrow and abnormalities in the Fas/FasL apoptotic pathway cause autoreactive B cells to escape into the periphery (12, 13); peripherally, serum BLyS/BAFF and APRIL levels are significantly elevated and positively correlated with disease activity, continuously sustaining B-cell survival through BAFF-R/BCMA receptors (14, 15). At the same time, excessive CD40-CD40L costimulatory signaling and weakened CTLA-4/PD-1 inhibitory signaling further disrupt immune homeostasis (16). On the other hand, pathogenic B-cell subsets such as IgD^−^CD27^−^ DN B cells and CD11c^+^T-bet^+^ ABCs are selectively expanded in connective tissue disease patients (such as SLE) (16, 17); under the regulation of IL-21 secreted by Tfh cells, these cells rapidly differentiate into plasma cells and produce cross-reactive autoantibodies such as anti-SSA, anti-CCP, and anti-U1RNP (18). Ultimately, B cells mediate pathogenic effects through multiple pathways: autoantibodies form immune complexes with antigens and deposit in multiple organs, activating the complement system and causing local damage; they enhance antigen presentation and amplify T-cell activation; and they secrete pro-inflammatory cytokines such as IL-6 and TNF-α, which cooperate with the type I interferon pathway and cytotoxic T cells to build a cascade inflammatory network. Through epitope spreading and cytokine imbalance, they promote the coexistence of two or more connective tissue diseases (19).

The coexistence of SLE with three or more autoimmune diseases is extremely rare. Multiple disease processes are intertwined, leading to highly overlapping clinical manifestations, which significantly increases the difficulty of differential diagnosis and makes early recognition a major challenge. Reviewing the patient’s disease course, when the initial symptom was a rash in 2021, although vulgar psoriasis and erythematosus lupus were clearly diagnosed, the possibility of subsequent overlap with additional autoimmune diseases was not further recognized. When the patient presented with seizures in 2022, she did not yet have clear multisystem involvement of SLE, and only high-titer ANA and strongly positive anti-nRNP/Sm antibodies were detected. Therefore, attributing the seizure to Sjögren’s syndrome-related CNS involvement was reasonable based on the available clinical data at that time. However, the patient was subsequently diagnosed with SLE at our center. Published literature indicates that cerebral venous thrombosis is a known thrombotic complication of SLE, with seizures as a common clinical manifestation. In contrast, cerebral venous thrombosis in isolated Sjögren’s syndrome is rare and mostly limited to case reports (20). A retrospective reassessment of the seizure episode from three years ago suggests that the possibility of lupus encephalopathy cannot be excluded, rather than considering it as solely a central nervous system event of Sjögren’s syndrome. Therefore, for patients with an autoimmune background or suspicious clues, it is essential to establish a long-term, systematic follow-up and monitoring system in order to promptly capture new symptoms and serological changes, thereby revising the diagnosis and adjusting treatment as early as possible. This is crucial for improving the prognosis of such complex patients.

The treatment of this patient reflects the strategic judgment required in managing complex autoimmune overlap syndrome: prioritizing life-threatening organ damage while coordinating control of multisystem manifestations. In overlap syndrome with extensive multisystem involvement, dual therapy with glucocorticoids and conventional immunosuppressants often provides insufficient immunosuppression and fails to achieve adequate disease control. Rituximab, though indicated for refractory autoimmune diseases, carries risks of hypogammaglobulinaemia and severe infections. Calcineurin inhibitors are effective against lupus nephritis but have limited efficacy against myopathy and central nervous system lesions. Given these considerations, we adopted a triple regimen of glucocorticoids, cyclophosphamide, and telitacicept in this case. By targeting distinct immune pathways, this regimen synergistically suppresses autoimmune inflammation, protects target organs, and facilitates glucocorticoid tapering. In the early treatment phase, for the two critical conditions of active lupus nephritis and immune-mediated necrotizing myopathy, an intensified immunosuppressive regimen of high-dose methylprednisolone pulse therapy combined with cyclophosphamide and telitacicept was used, which is the cornerstone for rapidly controlling acute severe immune storm. However, treatment of overlap syndrome still faces core challenges: achieving treatment targets for multiple diseases and long-term control while avoiding risks such as infection caused by excessive immunosuppression. Abnormal B-cell activation and excessive autoantibody production are the common core mechanisms driving such overlap lesions; therefore, treatment strategies targeting B-cell depletion or inhibition of activation have become key directions for restoring immune balance and improving clinical symptoms. As the world’s first biologic capable of simultaneously targeting B-lymphocyte stimulator (BLyS/BAFF) and proliferation-inducing ligand (APRIL), telitacicept suppresses the development and survival of plasma cells and mature B cells through dual blockade (21), effectively reducing SLE activity and controlling nephropathy progression (22). In addition, studies have confirmed that telitacicept has clear efficacy and good safety in the treatment of connective tissue diseases such as SS and RA (23). Notably, its therapeutic value in overlap syndrome has also received preliminary clinical evidence: case reports showed that symptoms improved significantly after telitacicept treatment in 3 overlap patients with myasthenia gravis complicated by Sjögren’s syndrome/SLE (24), and another complex overlap case of Sjögren’s syndrome complicated by rheumatoid arthritis, autoimmune hepatitis, and renal tubular acidosis also achieved remarkable efficacy after telitacicept treatment (25). This patient received triple immunosuppressive therapy: during the intensive phase, glucocorticoid pulse therapy was combined with cyclophosphamide and telitacicept; during the stable phase, low-dose glucocorticoids and mycophenolate mofetil were adjusted for long-term maintenance therapy with telitacicept. Ultimately, the multiple autoimmune diseases and systemic damage dominated by SLE were effectively controlled.

## Conclusion

Treatment of overlap syndrome faces the difficult challenge of coordinating multiple therapeutic targets and balancing medication safety. The core pathogenesis lies in abnormal B-cell activation and excessive autoantibody production; therefore, targeted therapy against B cells and plasma cells is of great significance. Telitacicept, as the first dual-pathway inhibitor capable of simultaneously suppressing BLyS and APRIL, can regulate the activation of B cells and plasma cells and may have a promising application prospect in the treatment of complex overlap syndrome involving multiple autoimmune diseases. However, muscle biopsy was not performed in this case, immune-mediated necrotizing myopathy could only be regarded as a clinically suspected diagnosis supported by positive serological findings, which partly limits diagnostic accuracy and the evaluation of therapeutic response to targeted therapy. Meanwhile, the patient received high-dose glucocorticoids combined with cyclophosphamide during the intensive induction phase, and mycophenolate mofetil was added in the maintenance phase. Accordingly, the observed clinical improvement cannot be attributed solely to telitacicept. Although this case provides preliminary exploratory experience regarding telitacicept use in overlap syndrome, its definitive efficacy and safety remain to be further validated in larger-scale prospective studies.

## Data Availability

The raw data supporting the conclusions of this article will be made available by the authors, without undue reservation.
